# Role of Soluble Epoxide Hydrolase in Metabolism of PUFAs in Psychiatric and Neurological Disorders

**DOI:** 10.3389/fphar.2019.00036

**Published:** 2019-01-30

**Authors:** Kenji Hashimoto

**Affiliations:** Division of Clinical Neuroscience, Center for Forensic Mental Health, Chiba University, Chiba, Japan

**Keywords:** α-synuclein, cytochrome P450, dementia of Lewy bodies, depression, epoxy fatty acids, inflammation, Parkinson’s disease, stress resilience

## Abstract

Inflammation plays a key role in the pathogenesis of a number of psychiatric and neurological disorders. Soluble epoxide hydrolases (sEH), enzymes present in all living organisms, metabolize epoxy fatty acids (EpFAs) to corresponding 1,2-diols by the addition of a molecule of water. Accumulating evidence suggests that sEH in the metabolism of polyunsaturated fatty acids (PUFAs) plays a key role in inflammation. Preclinical studies demonstrated that protein expression of sEH in the prefrontal cortex, striatum, and hippocampus from mice with depression-like phenotype was higher than control mice. Furthermore, protein expression of sEH in the parietal cortex from patients with major depressive disorder was higher than controls. Interestingly, *Ephx2* knock-out (KO) mice exhibit stress resilience after chronic social defeat stress. Furthermore, the sEH inhibitors have antidepressant effects in animal models of depression. In addition, pharmacological inhibition or gene KO of sEH protected against dopaminergic neurotoxicity in the striatum after repeated administration of MPTP (1-methyl-4-phenyl-1,2,3,6-tetrahydropyridine) in an animal model of Parkinson’s disease (PD). Protein expression of sEH in the striatum from MPTP-treated mice was higher than control mice. A number of studies using postmortem brain samples showed that the deposition of protein aggregates of α-synuclein, termed Lewy bodies, is evident in multiple brain regions of patients from PD and dementia with Lewy bodies (DLB). Moreover, the expression of the sEH protein in the striatum from patients with DLB was significantly higher compared with controls. Interestingly, there was a positive correlation between sEH expression and the ratio of phosphorylated α-synuclein to α-synuclein in the striatum. In the review, the author discusses the role of sEH in the metabolism of PUFAs in inflammation-related psychiatric and neurological disorders.

## Introduction

Polyunsaturated fatty acids (PUFAs) are generally considered to be necessary for maintaining normal physiology (Jump, 2002; Bazinet and Layé, 2014; Layé et al., 2018). PUFAs are known to regulate both the structure and the function of neurons, glial cells, and endothelial cells in the brain (Bazinet and Layé, 2014; Layé et al., 2018). Importantly, PUFAs need to be provided by the diet since they cannot be produced in mammals. There are two main families (ω-3 and ω-6) of PUFAs. Linoleic acid, the predominant plant-derived dietary ω-6 PUFA, is a precursor of arachidonic acid (ARA). α-linolenic acid, the predominant plant-derived dietary ω-3 PUFA, is a precursor of eicosapentaenoic acid (EPA) and docosahexaenoic acid (DHA).

**Figure 1:**
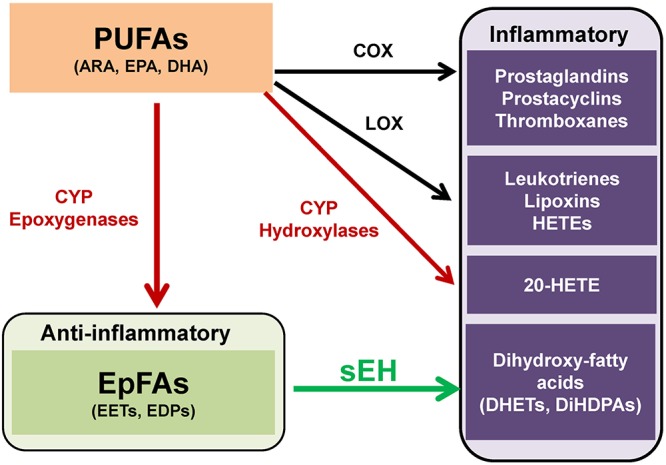
Overview of metabolism of polyunsaturated fatty acids (PUFAs). PUFAs such as arachidonic acid (ARA), eicosapentaenoic acid (EPA), and docosahexaenoic acid (DHA), are converted to prostaglandins, prostacyclins, and thromboxanes by cyclooxygenase (COX). PUFAs are also converted to leukotrienes, lipoxins, hydroxyeicosatetraenoic acids (HETEs) by lipoxygenase (LOX). Moreover, PUFAs are converted to hydroxyeicosatetraenoic acids (HETEs), including 20-hydroxyeicosatetraenoic acid (20-HETE), and epoxy fatty acids (EpFAs), including epoxyeicosatrienoic acids (EETs) and epoxydocosapentaenoic acids (EDPs), by cytochrome P450 (CYP) hydroxylases and CYP epoxygenases, respectively. EpFAs (e.g., EETs, EDPs) are converted to their corresponding 1,2-diols (e.g., dihydroxyeicosatrienoic acids (DHETs), dihydroxydocosapentaenoic acids [DiHDPAs]) by soluble epoxide hydrolase (sEH). (modified from Morisseau and Hammock, 2013 and Hashimoto, 2016).

Polyunsaturated fatty acids (PUFAs) are metabolized into bioactive derivatives by the main enzymes such as cyclooxygenases (COXs), lipoxygenases (LOXs), and cytochrome P450s (CYPs) (Imig and Hammock, 2009; Arnold et al., 2010; Imig, 2012, 2018; Morisseau and Hammock, 2013; Bazinet and Layé, 2014; Urquhart et al., 2015; Westphal et al., 2015; Figure 1). The COX pathway leads to the formation of prostaglandins, prostacyclines and thromboxanes, the LOX pathway leads to leukotrienes, lipoxins, and hydroxyl-eicosatetraenoic acids (HETEs). The CYP pathway leads to 20-HETE by CYP hydroxylases, and epoxy fatty acids (EpFAs) such as epoxy-eicosatrienoic acids (EETs) and epoxydocosapentaenoic acids (EDPs) by CYP epoxygenases (Figure 1).

In the review, the author would like to discuss the role of soluble epoxide hydrolase (sEH) in the CYP-mediated metabolism of PUFAs which might be involved in the pathogenesis of psychiatric and neurological disorders. Furthermore, we also refer to the clinical significance of sEH inhibitors for these disorders.

## Soluble Epoxide Hydrolase in CYP System

The CYP system is a superfamily of enzymes, which are involved in the metabolism of exogenous and endogenous compounds. The CYP in the eicosanoid pathway was first described in 1980 and is comprised of two enzymatic pathways such as hydroxylases and epoxygenases. The CYP isoforms metabolize a number of ω-3 and ω-6 PUFAs, including ARA, EPA and DHA into bioactive lipid mediators, termed eicosanoids (Imig and Hammock, 2009; Imig, 2012, 2018; Morisseau and Hammock, 2013; Urquhart et al., 2015; Westphal et al., 2015; Jamieson et al., 2017). The CYP system produces both the pro-inflammatory, terminally hydroxylated metabolite 20-HETE and the anti-inflammatory EpFAs, including EETs from ARA and EDPs from DHA (Figure 1).

In contrast, EpFAs such as EETs, and EDPs are rapidly metabolized by a number of pathways including the soluble epoxide hydrolase (sEH) (Imig and Hammock, 2009; Morisseau and Hammock, 2013). The sEH was first identified in the cytosolic fraction of mouse liver through its activity on epoxide containing substances such as juvenile hormone and lipid epoxides (Hammock et al., 1976; Gill and Hammock, 1980; Ota and Hammock, 1980). Human sEH is a 62 kDa enzyme composed of two domains separately by a short proline-rich linker (Harris and Hammock, 2013). The N-terminal domain has a phosphatase activity that hydrolyzes lipid phosphates, while the C-terminal domain has an epoxide hydrolase activity that converts epoxides to their corresponding diols (Newman et al., 2003). The human *EPHX2* gene codes for the sEH protein is widely expressed in a number of tissues, including the liver, lungs, kidney, heart, brain, adrenals, spleen, intestines, urinary bladder, placenta, skin, mammary gland, testis, leukocytes, vascular endothelium, and smooth muscle. Interestingly, the sEH protein is most highly expressed in the liver and kidney (Gill and Hammock, 1980; Newman et al., 2005; Imig, 2012).

Accumulating evidence suggests that EETs, EDPs and some other EpFAs have potent anti-inflammatory properties (Wagner et al., 2014, 2017; López-Vicario et al., 2015) which are implicated in the pathogenesis of a number of psychiatric and neurological disorders (Denis et al., 2015; Hashimoto, 2015, 2016, 2018; Gumusoglu and Stevens, 2018; Polokowski et al., 2018).

## Inflammation in Depression and sEH

Depression, one of the most common disorders in the world, is a major psychiatric disorder with a high rate of relapse. The World Health Organization (WHO) estimates that more than 320 million individuals of all ages suffer from depression (World Health Organization [WHO], 2017). Multiple lines of evidence demonstrate inflammatory processes in the pathophysiology of depression and in the antidepressant actions of the certain compounds (Dantzer et al., 2008; Miller et al., 2009, 2017; Raison et al., 2010; Hashimoto, 2015, 2016, 2018; Mechawar and Savitz, 2016; Miller and Raison, 2016; Zhang et al., 2016a,b, 2017b,a). Meta-analysis showed higher levels of pro-inflammatory cytokines in the blood of drug-free or medicated depressed patients compared to healthy controls (Dowlati et al., 2010; Young et al., 2014; Haapakoski et al., 2015; Eyre et al., 2016; Köhler et al., 2018). Collectively, it is likely that inflammation plays a key role in the pathophysiology of depression.

Several reports using meta-analysis demonstrated that ω-3 PUFAs could reduce depressive symptoms beyond placebo (Lin et al., 2010, 2017; Sublette et al., 2011; Mello et al., 2014; Grosso et al., 2016; Hallahan et al., 2016; Mocking et al., 2016; Sarris et al., 2016; Bai et al., 2018; Hsu et al., 2018). Dietary intake of ω-3 PUFAs is known to be associated with lower risk of depression. Importantly, EPA-rich ω-3 PUFAs could be recommended for the treatment of depression (Sublette et al., 2011; Mocking et al., 2016; Sarris et al., 2016). Importantly, brain EPA levels are 250-300-fold lower than DHA compared to about 4- (plasma), 5- (erythrocyte), 14- (liver), and 86-fold (heart) lower levels of EPA versus DHA (Chen and Bazinet, 2015; Dyall, 2015).

**Figure 2:**
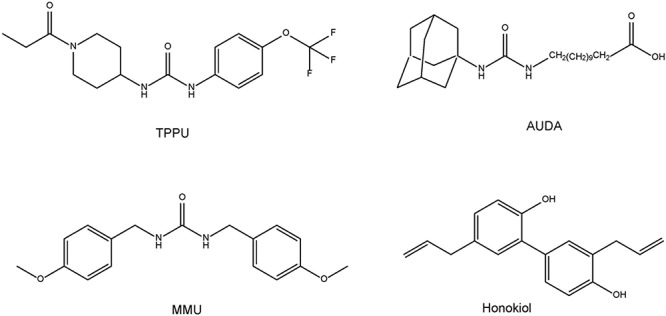
Chemical structure of sEH inhibitors TPPU, AUDA, MMU, and honokiol.

**Figure 3:**
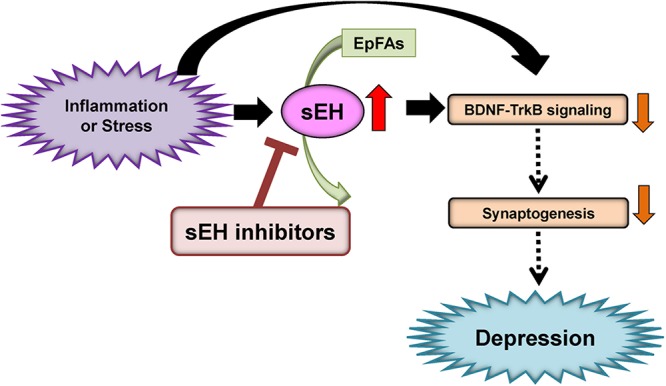
Proposed mechanism of the role of sEH in depression. Inflammation or stress can increase the expression of sEH in the brain, resulting in enhanced metabolism of anti-inflammatory PUFA epoxides (EpFAs). Subsequently, increased expression of sEH can decrease BDNF-TrkB signaling and synaptogenesis, leading to depressive symptoms. The sEH inhibitors may have antidepressant actions in depressed patients. (modified from Hashimoto, 2016).

Given the role of inflammation in depression, it is likely that sEH might contribute to the pathophysiology of depression. A single injection of lipopolysaccharide (LPS) is known to produce depression-like phenotypes in rodents after sickness behaviors (Dantzer et al., 2008; Zhang et al., 2014, 2016a, 2017b; Ma et al., 2017; Yang et al., 2017). Ren et al. (2016) reported that the sEH inhibitor TPPU [1-(1-propionylpiperidin-4-yl)-3-(4-(trifluoromethoxy)phenyl)urea] (Figure 2) conferred prophylactic and antidepressant effects in the LPS-induced inflammation model of depression while the current antidepressants showed no therapeutic effects in this model (Zhang et al., 2014). Chronic social defeat stress (CSDS) model of depression is widely used as an animal model of depression (Nestler and Hyman, 2010; Golden et al., 2011; Yang et al., 2015, 2017, 2018). Pretreatment with TPPU before social defeat stress showed resilience to CSDS. In addition, TPPU showed rapid antidepressant effects in susceptible mice after CSDS (Ren et al., 2016). Interestingly, the sEH KO mice showed stress resilience to repeated social defeat stress. Increased brain-derived neurotrophic factor (BDNF) and its receptor TrkB signaling in the prefrontal cortex and hippocampus of the KO mice might be responsible for stress resilience (Ren et al., 2016). Furthermore, repeated treatment with TPPU for 7 days increased interaction time of socially defeated mice in a CSDS model, and improvement by TPPU was blocked by TrkB antagonist K252a (Wu et al., 2017), suggesting a role of BDNF-TrkB signaling in TPPU’s beneficial effects. Interestingly, higher protein levels of sEH were shown in the brain regions of mice with a depression-like phenotype in the inflammation and CSDS models, suggesting that increased levels of sEH may play a role in depression-like phenotypes in rodents (Ren et al., 2016). We found higher sEH protein levels in the parietal cortex (Brodmann area 7) from patients with major depressive disorder, pointing to a possible role for increased sEH levels in depression (Ren et al., 2016). Taken together, this study highlights a key function for sEH in the pathophysiology of depression, and for its inhibitors as potential therapeutic or prophylactic drugs for depression (Hashimoto, 2016; Ren et al., 2016; Swardfager et al., 2018; Figure 3).

A study using euthymic patients with a history of major depressive disorder with seasonal depression showed changes in CYP- and sEH-derived eicosanoids in patients with winter depression (Hennebelle et al., 2017). The ω-6 derived sEH product 12,13-DiHOME [12,13-dihydroxy-9-octadecenoic acid] increased in winter depression. Total 14,15-EpETE [14,15-epoxy-5Z,8Z,11Z,17Z-eicosatetraenoic acid], a sEH substrate, as well as sEH-derived free 14,15-DiHETrE [14,15-dihydroxy-5Z,8Z,11Z- eicosatrienoic acid], decreased during winter compared to summer-fall, while sEH-derived total 7,8-DiHDPE [7,8-dihydroxy-4Z,10Z,13Z,16Z,19Z-docosapentaenoic acid], total 19,20-DiHDPE [19,20-dihydroxy-4Z,7Z,10Z,13Z,16Z-docosapentaenoic acid], and total 12,13-DiHOME [12,13-dihydroxy-9Z-octadecenoic acid] were increased during winter. These findings suggest that seasonal shifts in ω-6 and ω-3 PUFAs metabolism mediated by sEH may underlie inflammatory states in symptomatic depression with seasonal pattern (Hennebelle et al., 2017). Given the crucial role of sEH in the metabolism of ω-3 PUFAs, ω-3 PUFAs such as EPA in combination with a sEH inhibitor would be a novel therapeutic approach for depression (Figure 3).

## Eating Disorders and ADHD

Anorexia nervosa (AN) is a serious eating disorder characterized by the persistent restriction of energy intake, intense fear of gating weight, and distribution in self-perceived weight or shape. The Epoxide Hydrolase 2 (*EPHX2)* gene was found to harbor several common and rare risk variants for AN (Scott-Van Zeeland et al., 2014). Subsequently, the patients with AN show elevated plasma levels of ω-3 PUFAs (ARA, EPA, DHA) compared to controls (Shih et al., 2016). Interestingly, 15,16-DiHODE [15,16-dihydroxy-9Z,12Z-octadecadienoic acid]/15,16-EpODE [15,16-epoxy-9Z,12Z-octadecadienoic acid] ratio derived from ARA and 19,20-DiHDPE [19,20-dihydroxy-4Z,7Z,10Z,13Z,16Z- docosapentaenoic acid]/19,20-EpDPE [19,20-epoxy-4Z,7Z,10Z,13Z,16Z-docosapentaenoic acid] ratio derived from DHA in AN patients were higher than controls, suggesting a higher *in vivo* sEH activity, concentration, or efficiency in AN (Shih et al., 2016; Shih, 2017). These data suggest the role of EPHX2-associated eicosanoid dysregulations in AN. Collectively, sEH inhibitors might be potential therapeutic drugs for AN (Shih et al., 2016; Shih, 2017).

Attention deficit hyperactivity disorder (ADHD) is one of the most common psychiatric disorders affecting children. Symptoms of ADHD include inattention, hyperactivity and impulsivity. However, biological mechanisms underlying ADHD remain unknown. A meta-analysis shows that children and youth with ADHD have elevated ratios of both blood ω-6/ω-3 PUFAs compared to controls (LaChance et al., 2016), suggesting an elevated ω-6/ω-3, and more specifically ARA/EPA ratio may represent the underlying disturbance in essential PUFAs levels in patients with ADHD. A recent meta-analysis shows that children and adolescents with ADHD have lower levels of DHA, EPA, and total ω-3 PUFAs (Chang et al., 2018). Furthermore, supplementation of ω-3 PUFAs, particularly with high doses of EPA, was modestly effective in the treatment of ADHD (Bloch and Qawasmi, 2011; Chang et al., 2018). Collectively, it is of great interest to study whether blood levels of EpFAs and their corresponding diols are altered in the patients with ADHD. Furthermore, it is also interesting to investigate the role of sEH in the pathogenesis of ADHD since there are no reports showing the role of sEH in ADHD.

## Inflammation in Parkinson’s Disease and sEH

Parkinson’s disease (PD) is the second most common neurodegenerative disease after Alzheimer’s disease. Although the precise pathogenesis of PD is unknown, the pathological hallmark of PD involves the progressive loss of dopaminergic neurons in the substantia nigra (SN) (Kalia and Lang, 2015; Ascherio and Schwarzschild, 2016). In addition, the deposition of aggregates of α-synuclein, termed Lewy bodies, is evident in multiple brain regions of patients from PD and dementia with Lewy bodies (DLB) (Spillantini et al., 1997). There are, to date, no agents with a disease-modifying or neuroprotective indication for PD has been approved (Dehay et al., 2015). Interestingly, it is noteworthy that PD or DLB patients have depressive symptoms (Cummings, 1992; Takahashi et al., 2009; Goodarzi et al., 2016; Schapira et al., 2017), indicating that management of depression in these patients is also important. Therefore, the development of new drugs possessing disease-modifying and/or neuroprotective properties is unmet medical need.

**Figure 4:**
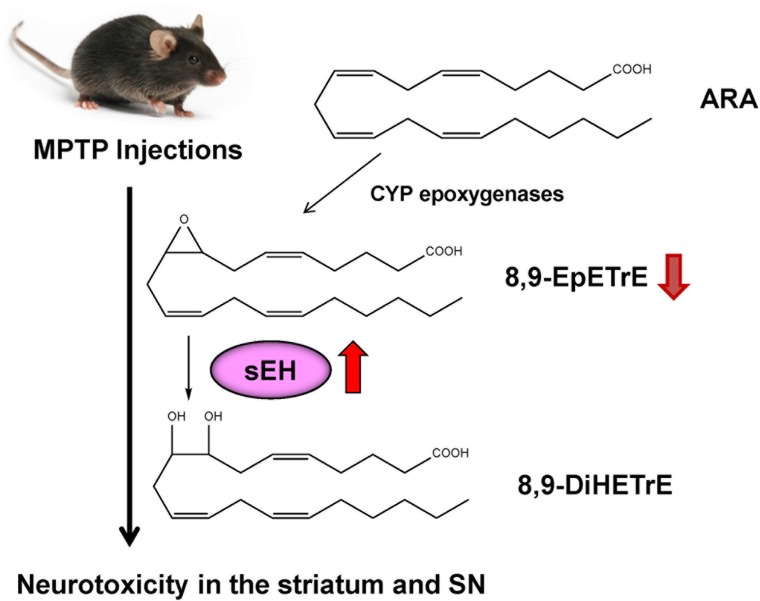
Possible mechanism of role of sEH in the MPTP-induced neurotoxicity. 8,9-EpETrE is prepared from ARA by CYP epoxygenases, and it is metabolized by sEH into 8,9-DiHETrE. Repeated MPTP injections into mice caused increased sEH expression in the striatum, resulting the reduction of anti-inflammatory 8,9-EpETrE in the striatum. Finally, these events cause dopaminergic neurotoxicity in the striatum and SN. Pharmacological inhibition or knock-out of sEH could protect against MPTP-induced neurotoxicity in the striatum and SN.

ω-3 PUFAs appear to be neuroprotective for several neurological disorders. It is reported that dietary intake of PUFAs is associated with lower risk of PD (Kamel et al., 2014; Seidl et al., 2014). MPTP (1-methyl-4-phenyl-1,2,3,6-tetrahydropyridine)-induced neurotoxicity in the striatum and SN has been widely used as an animal model of PD (Sedelis et al., 2001; Jackson-Lewis and Przedborski, 2007). A diet rich in EPA diminished MPTP-induced hypokinesia in mice and ameliorated procedural memory deficit (Luchtman et al., 2012). Recently, we reported that MPTP-induced neurotoxicity [e.g., loss of dopamine transporter (DAT), loss of tyrosine hydrolase (TH)-positive cells, increased endoplasmic reticulum (ER) stress] in the striatum and SN was attenuated after subsequent repeated oral administration of TPPU (Ren et al., 2018). MPTP-induced loss of TH-positive cells in the SN is also attenuated by pretreatment with another sEH inhibitor, AUDA [12-(((tricyclo(3.3.1.13,7)dec-1-ylamino)carbonyl)amino)-dodecanoic acid] (Figure 2), although posttreatment with AUDA did not attenuate MPTP-induced neurotoxicity (Qin et al., 2015). Furthermore, deletion of the sEH gene protected against MPTP-induced neurotoxicity in the mouse striatum (Huang et al., 2018; Ren et al., 2018), while overexpression of sEH in the striatum significantly enhanced MPTP-induced neurotoxicity (Ren et al., 2018). Moreover, the expression of the sEH protein in the striatum from MPTP-treated mice was significantly higher than control group. Interestingly, there was a positive correlation between sEH expression and phosphorylation of α-synuclein in the striatum, suggesting that sEH may play a role in the phosphorylation of α-synuclein in the mouse striatum (Ren et al., 2018). Oxylipin analysis showed reduced levels of 8,9-epoxy-5Z,11Z,14Z-eicosatrienoic acid (8,9-EpETrE) prepared from ARA in the striatum of MPTP-treated mice, suggesting increased activity of sEH in this region (Figure 4).

Deposition of α-synuclein has been shown in multiple brain regions of PD and DLB patients (Spillantini et al., 1997). Interestingly, the high levels of DHA in brain areas containing α-synuclein in PD patients may support the possible interaction between α-synuclein and DHA (Fecchio et al., 2018). Protein levels of sEH in the striatum from DLB patients were significantly higher than those of the controls, whereas protein levels of DAT and TH in the striatum from DLB patients were significantly lower than those of controls (Ren et al., 2018). Furthermore, the ratio of phosphorylated α-synuclein to α-synuclein in the striatum from DLB patients was significantly higher than that of controls (Ren et al., 2018). Interestingly, there was a positive correlation between sEH levels and the ratio of phosphorylated α-synuclein to α-synuclein in all subjects (Ren et al., 2018). Collectively, it is likely that increased sEH and resulting increase in phosphorylation of α-synuclein may play a role in the pathogenesis of PD.

**Figure 5:**
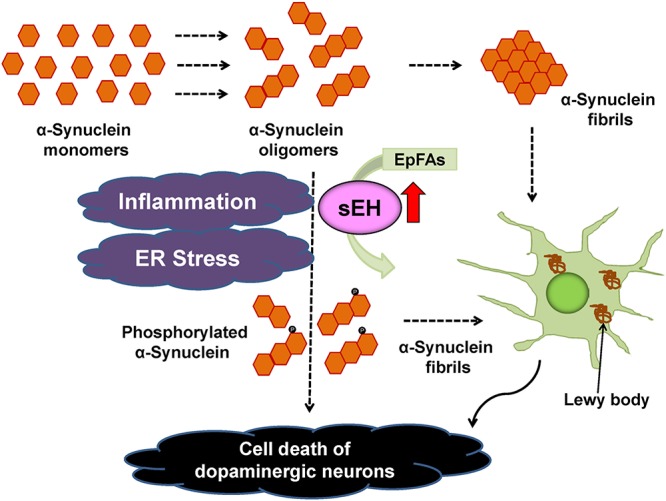
Proposed mechanism of the role of sEH in the pathogenesis of PD and DLB. Inflammation and ER stress can increase the expression of sEH in the striatum, resulting in enhanced metabolism of anti-inflammatory EpFAs, leading to increased phosphorylation of α-synuclein (Ren et al., 2018). The sEH inhibitors may prevent the progression of aggregation of phosphorylated α-synuclein in the brain.

The PARK2 is one of the familial forms of PDs caused by a mutation in the *PARKIN* gene (Imaizumi et al., 2012). In addition, the expression of *EPHX2* mRNA in human PARK2 iPSC-derived neurons was higher than that of healthy control group. Treatment with TPPU protected against apoptosis in human PARK2 iPSC-derived neurons (Ren et al., 2018). These findings suggest that increased activity of sEH in the striatum plays a key role in the pathogenesis of neurological disorders such as PD and DLB although common polymorphisms within *EPHX2* do not appear to be important risk factors for PD (Farin et al., 2001). Accumulation of aggregated α-synuclein is the pathological hallmark of PD and DLB although its precise role is not understood. Our data suggest a possible interaction between phosphorylation of α-synuclein and sEH expression in the striatum from DLB patients. Taken all together, it is likely that sEH could represent a promising therapeutic target for α-synuclein-related neurological disorders such as PD and DLB (Borlongan, 2018; Ren et al., 2018; Figure 5). In addition, there are also several approaches (e.g., a small-interfering RNA, immunotherapies, enhancement of autophagy) to reduce α-synuclein production (Stoker et al., 2018).

## Conclusion Remarks and Future Perspective

Many patients with depression become chronically ill, with several relapses or later recurrences, following initial short-term improvement or remission. Relapses occur at a rate of over 85 percent within a decade of an index depressive episode (Forte et al., 2015; Sim et al., 2015). Therefore, the prevention of relapse and recurrence is important in the management of depression. Taken together, it seems that sEH inhibitors could be prophylactic drugs to prevent or minimize relapses triggered by inflammation and/or stress in remitted patients with depression (Hashimoto, 2016; Ren et al., 2016). In addition, given the comorbidity of depressive symptoms in PD or DLB patients (Cummings, 1992; Takahashi et al., 2009; Goodarzi et al., 2016; Schapira et al., 2017), it is also likely that sEH inhibitors may serve as prophylactic drugs to prevent the progression of PD or DLB in patients.

Some natural compounds with sEH inhibitory action were reported. MMU [1,3-bis (4-methoxybenzyl)urea](Figure 2), the most abundant (45.3 μg/g dry root weight from the plant *Pentadiplandra brazzeana*), showed an IC_50_ of 92 nM via fluorescent assay and a *K*i of 54 nM via radioactivity-based assay on human sEH (Kitamura et al., 2015). MMU is about 8-fold more potent than previously reported natural product sEH inhibitor honokiol (Lee et al., 2014; Kitamura et al., 2015; Figure 2). These findings may explain partly the pharmacological mechanisms of the traditional medicinal use of the root of *P. brazzeana.* Therefore, it is of interest to study whether the use of the root of *P. brazzeana* has beneficial effects in patients with psychiatric and neurological disorders.

Another topic is the systemic anti-inflammatory effects of sEH inhibition or genetic disruption (Liu et al., 2012; Shahabi et al., 2014). Therefore, it is possible that systemic sEH inhibition may play a role in the beneficial effects in CNS disorders through systemic anti-inflammatory actions of sEH inhibition although further study on the role of systemic anti-inflammation effects of sEH inhibition is needed. It is also suggested that a paracrine role of EET signaling is responsible for a lot of the beneficial effects of EETs (Spector, 2009; Imig, 2016). Therefore, it is possible that up-regulation of sEH, which results in decreased paracrine EET signaling that exasperates the disease state although further study on the role of paracrine role of EETs and sEH is needed.

In conclusion, considering the role of sEH in the metabolism of EpFAs (e.g., EETs, EDPs), treatment of ω-3 PUFAs in combination with a sEH inhibitor could represent a novel therapeutic approach for psychiatric and neurological disorders. This approach may well bridge the currently unmet medical needs for these CNS disorders.

## Author Contributions

The author confirms being the sole contributor of this work and has approved it for publication.

## Conflict of Interest Statement

The author declares that the research was conducted in the absence of any commercial or financial relationships that could be construed as a potential conflict of interest.
